# Diet-Induced Obesity Disturbs Microglial Immunometabolism in a Time-of-Day Manner

**DOI:** 10.3389/fendo.2019.00424

**Published:** 2019-06-26

**Authors:** Irina V. Milanova, Martin J. T. Kalsbeek, Xiao-Lan Wang, Nikita L. Korpel, Dirk Jan Stenvers, Samantha E. C. Wolff, Paul de Goede, Annemieke C. Heijboer, Eric Fliers, Susanne E. la Fleur, Andries Kalsbeek, Chun-Xia Yi

**Affiliations:** ^1^Department of Endocrinology and Metabolism, Amsterdam University Medical Center, University of Amsterdam, Amsterdam, Netherlands; ^2^Laboratory of Endocrinology, Amsterdam University Medical Center, Amsterdam Gastroenterology & Metabolism, University of Amsterdam, Amsterdam, Netherlands; ^3^Endocrine Laboratory, Department of Clinical Chemistry, Amsterdam University Medical Center, Amsterdam Gastroenterology & Metabolism, Vrije Universiteit Amsterdam, Amsterdam, Netherlands; ^4^Netherlands Institute for Neuroscience, Royal Netherlands Academy of Arts and Sciences, Amsterdam, Netherlands

**Keywords:** microglia, immunometabolism, neuroinflammation, diet-induced obesity, high-fat diet, daily rhythms

## Abstract

**Background:** Disturbance of immunometabolic signaling is a key process involved in the progression of obesity. Microglia—the resident immune cells in the brain, initiate local immune responses. It is known that hypercaloric diets lead to microglial activation. Previously, we observed that hypothalamic microglial cells from mice fed high-fat diet (HFD) lose their day/night rhythm and are constantly activated. However, little is known about daily rhythmicity in microglial circadian, immune and metabolic functions, either in lean or obese conditions. Therefore, we hypothesized that HFD disturbs microglial immunometabolism in a day/night-dependent manner.

**Methods:** Obesity was induced in Wistar rats by feeding them HFD *ad libitum* for the duration of 8 weeks. Microglia were isolated from HFD- and chow-fed control animals at six time points during 24 h [every 4 h starting 2 h after lights on, i.e., Zeitgeber Time 2 (ZT2)]. Gene expression was evaluated using quantitative RT-PCR. JTK_Cycle software was used to estimate daily rhythmicity. Statistical analysis was performed with two-way ANOVA test.

**Results:** Consumption of the obesogenic diet resulted in a 40 g significantly higher body weight gain in week 8, compared to chow diet (*p* < 0.0001), associated with increased adiposity. We observed significant rhythmicity of circadian clock genes in microglia under chow conditions, which was partially lost in diet-induced obesity (DIO). Microglial immune gene expression also showed time-of-day differences, which were disrupted in HFD-fed animals. Microglia responded to the obesogenic conditions by a shift of substrate utilization with decreased glutamate and glucose metabolism in the active period of the animals, and an overall increase of lipid metabolism, as indicated by gene expression evaluation. Additionally, data on mitochondria bioenergetics and dynamics suggested an increased energy production in microglia during the inactive period on HFD. Finally, evaluation of monocyte functional gene expression showed small or absent effect of HFD on peripheral myeloid cells, suggesting a cell-specific microglial inflammatory response in DIO.

**Conclusions:** An obesogenic diet affects microglial immunometabolism in a time-of-day dependent manner. Given the central role of the brain in energy metabolism, a better knowledge of daily rhythms in microglial immunometabolism could lead to a better understanding of the pathogenesis of obesity.

## Introduction

Arising evidence highlights the disturbed interaction between immunity and metabolism as a key player in the pathogenesis of obesity (1–3). Immune cell function is highly dependent on metabolic adaptation of the immune cells, allowing for abrupt shifts in energy utilization, thus promoting either a resting or an activated state (4). Moreover, distinct immune cell populations show specific metabolic patterns, modulating their functional properties (4). In the brain, microglia are involved in maintaining brain homeostasis by surveying the environment, sensing invading pathogens and phagocyting dead neurons, and cellular debris, thus eliciting an innate immune response (5, 6). Microglial metabolic reprogramming is associated with polarization to pro- or anti-inflammatory state, which involves both functional and phenotypic plasticity (7, 8). It has been shown that hypercaloric environment induces a proinflammatory response in the hypothalamus via NF-kB and toll-like receptor activation, leading to disturbed energy homeostasis (9–13). This could be due to hypothalamic microglial activation as seen in rodents fed an obesogenic diet (14–17). We observed that under physiological conditions in mice, microglial cells exert their function in a strict time-of-day manner with higher activity during the dark, active phase, compared to the light, sleep phase (18). However, this day-night rhythm was abolished in animals fed an obesogenic, high-fat diet (HFD), suggesting an interaction of diet content and daily rhythms. Indeed, recent evidence suggest an involvement of circadian function in the progression of obesity (19, 20). It is well-known now that a master circadian clock in mammals generates daily rhythms in behavioral, physiological, and hormonal processes to allow adaptation to daily environmental changes, thus optimizing metabolic function to the time of day (21). However, little is known about daily rhythms in microglial function. Therefore, we performed a detailed investigation of daily rhythmicity in microglial immunometabolism in lean and obese rats. As mentioned earlier, many studies have focused on hypothalamic microglial inflammatory response due to the clear relation between the hypothalamus and energy homeostasis. Here, we chose to evaluate cortical microglial activation, to expand on available knowledge on microglial immunometabolism in obesity outside of the hypothalamus.

**Figure 1:**
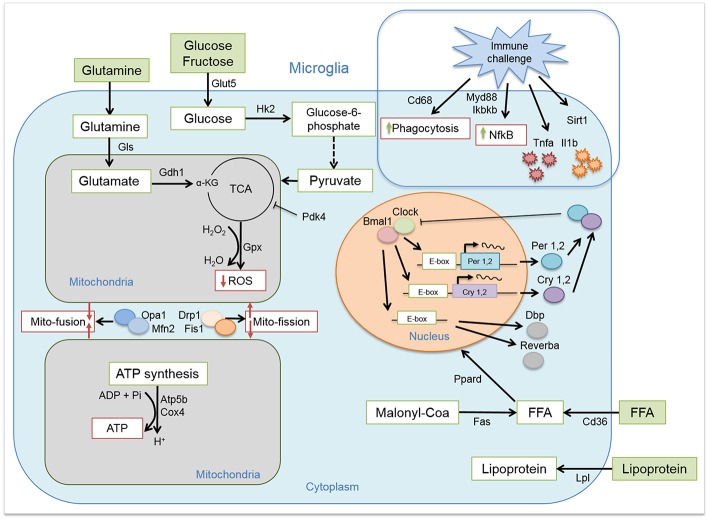
Microglial circadian, immune, metabolic, and mitochondrial profile. Schematic representation of the pathways and/or functions tested on microglial cells from chow- and HFD-fed rats.

We induced obesity with HFD for the duration of 8 weeks in rats and evaluated the expression of key clock genes involved in maintaining circadian rhythms (Figure 1). Microglial cells, as many other immune cells, have a high metabolic demand (22). Therefore, we also evaluated the expression of key genes involved in microglial glucose, lipid, and glutamate metabolism. As higher activity and substrate utilization require higher energy production we also assessed the state of mitochondria bioenergetics and dynamics in response to either healthy or obesogenic diet. The immune state of the cells was studied by evaluating cytokine production and phagocytosis (Figure 1). Our results showed time-of-day disturbances in microglial circadian and inflammatory functions in the obesogenic conditions, accompanied with changes in substrate utilization and energy production. We compared these data to monocytes, isolated from the same animals, to evaluate the state of peripheral myeloid cells in a hypercaloric environment. We observed a small effect of HFD on monocyte function, suggesting a microglia-specific response to hypercaloric intake. These results shed further light on microglial time-of-day innate immunometabolism in health and obesity.

## Methods

### Animals

Seventy-two male Wistar rats (Charles River, Germany) were group housed on a 12-h-light/12-h-dark cycle [lights on at 7:00 am; Zeitgeber time zero (ZT0)] at 22 ± 2°C with access to food and water *ad libitum*. Obesity was induced for the duration of 8 weeks, with a diet containing 60 kcal% fat and 20 kcal% carbohydrates (HFD, 5.24 kcal/g, D12492, Research Diets Inc.). Control animals were fed a standard chow diet (3.1 kcal/g, 2018, Teklad diets, Invigo). Body weight was monitored once per week, and food intake twice per week. All studies were approved by the Animal Ethics Committee of the Royal Dutch Academy of Arts and Sciences (KNAW, Amsterdam) and performed according to the guidelines on animal experimentation of the Netherlands Institute for Neuroscience (NIN, Amsterdam).

### Microglia/Monocyte Isolation and Plasma Collection

Animals were sacrificed at six time points during 24 h (every 4 h starting at ZT2) by euthanasia with 60% CO_2_/40% O_2_, followed by decapitation. Perirenal white adipose tissue (pWAT) was dissected for evaluation of fat mass gain, evaluating the amount of pWAT in grams weight. Microglial cells from cerebral cortex were isolated for gene expression analysis using the Percoll isopycnic isolation, as it provides a high cell number (23). Briefly, brains were mechanically homogenized with RPMI 1640 medium (Ref.: 11875-093, Gibco™) and filtered through 70 μm cell strainer (Ref.: 431751, Corning®) in a 15 mL Falcon tube. Brain homogenate was centrifuged for 5′ (380 g, 4°C). Pellets were resuspended with 7 mL RPMI medium and mixed with 100% Percoll solution [for 10 mL: 9 mL Percoll® stock (Ref.: 17-5445-01, GE Healthcare, Sigma-Aldrich®) with 1 mL 10x HBSS (Ref.: 14185-045, Gibco™)]. The cell suspension was layered slowly on 70% Percoll solution [for 10 mL: 7 mL 100% Percoll solution with 3 mL 1x HBSS (Ref.: 14175-053, Gibco™)] and centrifuged for 30′ (500 g, 18°C, break 1/0). Cell debris on the surface was discarded and fuse interphase, containing microglial cells were collected in 8 mL 1x HBSS, followed by centrifuging for 7′ (500 g, 18°C, break 9/9). Supernatant was discarded and the microglial cell pellet was used directly for RNA extraction.

During decapitation trunk blood was collected for measurement of different parameters. Briefly, blood was collected in 50 mL Falcon tubes, containing 0.5 M EDTA (ethylenediaminetetraacetic acid). Blood was filtered through a 70 μm cell strainer in a 15 mL Falcon tube and separated for monocyte isolation. For plasma collection, 2 mL blood was centrifuged for 15′ (4,000 rpm, 4°C, break 9/9). Plasma was collected in a new tube and stored at −80°C until usage. For monocyte isolation, 30 mL lysis buffer (containing 1 × ACK; 155 mM NH_4_Cl; 10 mM KHCO_3_; 0.1 mM EDTA) was added to ~3 mL blood and vortexed gently, followed by incubation at RT for 10–15′. The cell suspension was centrifuged for 5′ (200 g, RT, 9/9 break), supernatant was discarded and cells were resuspended in 2 mL PBS-FBS (PBS containing 1% FBS). The new cell suspension was again centrifuged for 5′ (200 g, RT, 9/9 break), supernatant was discarded and cells were resuspended in 0.5 mL PBS-FBS. The cell suspension was added to 4.5 mL RPMI medium and layered slowly on 5 mL Ficoll® (Ref.: 17-1440-02, GE Healthcare, Sigma-Aldrich®), followed by centrifuging for 30′ (400 g, 20°C, break 1/1). The fuse interphase, containing monocytes, was collected in 8 mL 1x HBSS, followed by centrifuging for 5′ (200 g, RT). Supernatant was discarded and the monocyte pellet was used for RNA extraction.

### Real-Time PCR

For gene expression analysis, RNA from microglial cells and monocytes was extracted using the RNeasy Micro Kit (Cat No. 74004, Qiagen®) according to the manufacturer's guidelines. RNA was quantified by spectrophotometry at 260 nm (DS 11; Denovix). RNA was reverse transcribed using Transcriptor First Strand cDNA Synthesis Kit (04897030001; Roche) according to the manufacturer's guidelines. Levels of mRNA for *Tnfa, Bmal1, Per1, Per2, Cry1, Cry2, Dbp, Reverba, Clock, Gls, Gdh, Gpx1, Cd36, Fas, Lpl1, Opa1, Mfn2, Fis1, Drp1, Pdk4, Ppard, Ikbkb, Cd68, Il1b, Cox4, Atp5b, Atp5g, Hk2, Glut5, Myd88, Sirt1, Hprt* (internal control), and *bactin* (internal control) were measured by semiquantitative real-time PCR on a LightCycler LC480 (Roche), using the SensiFAST SYBR® No-ROX Kit (BIO-98020, GC-Biotech) according to the manufacturer's guidelines. Expression levels of all genes were normalized to the geometric mean of the internal controls. Primer sequences (see Table S1) were designed using the Basic Local Alignment Search Tool (BLAST) from the National Center for Biotechnology Information (NCBI). Primers were purchased from Sigma-Aldrich® and validated by melt curve analysis and DNA band size and/or purity on agarose gel electrophoresis (data not shown).

### Glucose, Insulin, and Non-esterified Fatty Acids (NEFA) Measurements in Plasma

Plasma glucose concentrations were measured using the Glucose GOD-PAP kit (Ref. 80009, Biolabo S.A.S.), following the manufacturer's guidelines. Absorbance of colored samples, proportional to glucose concentration, was measured at 500 nm with Varioskan® Flash spectral scanning multimode reader (Version 40053; Thermo Scientific). Insulin concentrations were measured using Rat Insulin Radioimmunoassay (RIA) Kit (RI-13K; Millipore, Merck), according to the manufacturer's guidelines. Non-esterified fatty acids (NEFA) concentration in plasma was measured using the NEFA HR(2) reagents (R1 set, Ref. 434-91795; R2 set, Ref. 436-91995; Standard, Ref. 270-77000, Wako Chemicals GmbH) following the adjusted protocol from the Mouse Metabolic Phenotyping Centers [https://www.mmpc.org/shared/document.aspx?id=196&docType=Protocol]. Within-run variations for all measurements fall in the range suggested by the manufacturers.

### Statistical Analyses

**Figure 2:**
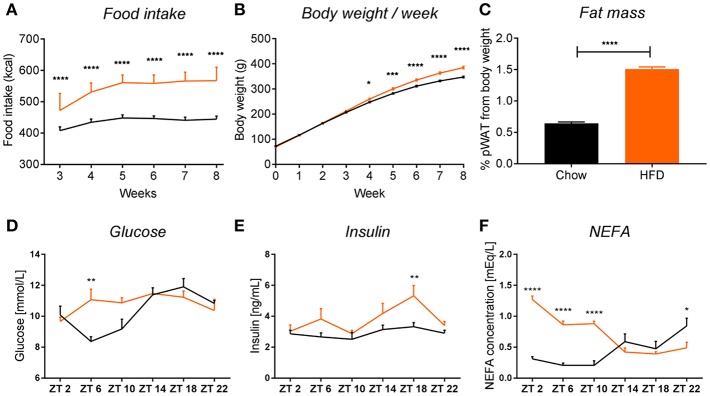
HFD intake leads to obesity in rats. HFD (orange) leads to an increase in food intake **(A)** (*p*_int_ < 0.0001, *p*_time_ < 0.0001, *p*_diet_ < 0.0001), body weight **(B)** (*p*_int_ < 0.0001, *p*_time_ < 0.0001, *p*_diet_ = 0.0003), and fat mass gain, seen as 2-fold increase in perirenal white adipose tissue **(C)** in rats, compared to chow-fed controls (black). Plasma measurements in non-fasted HFD-fed animals show an increase in glucose at ZT6 **(D)** (*p*_int_ = 0.0032, *p*_time_ = 0.0003, *p*_diet_ = 0.0874), increased insulin at ZT18 **(E)** (*p*_int_ = 0.2802, *p*_time_ = 0.0042, *p*_diet_ = 0.0006), and significant increase in non-esterified fatty acids during the light phase **(F)** (*p*_int_ < 0.0001, *p*_time_ = 0.0006, *p*_diet_ < 0.0001). Data are presented as means ± SEM. Statistical significance was determined using Two-way ANOVA **(A,B,D–F)** or unpaired *t*-test **(C)**. Effect of diet within each time point was evaluated with Sidak's multiple comparison test **(A,B,D–F)** (*p < 0.05; **p < 0.01, ***p < 0.001, ****p < 0.0001).

All results are expressed as mean ± SEM. Statistical analyses were performed using Graph-Pad PRISM (version 7.03, GraphPad Software, Inc.) and JTK_Cycle software (24). Two-way ANOVA analysis was used for effects of *Diet, Time*, (ZT) and *Interaction*. Unpaired *t*-tests were used to evaluate the effect of diet for each time point, unless stated otherwise. Sidak's multiple comparison test was used to compare the effect of diet for the food intake, body weight gain, and plasma measurements data (Figures 2A,B,D–F). One-way ANOVA analysis was used to assess the effect of *Time* for the chow and HFD groups separately. JTK_Cycle analysis *p*-values were obtained by fitting the data on a curve with fixed 24 h period. Results were considered statistically significant when *p* < 0.05.

## Results

### HFD Intake Induces Obesity in Rats

We observed that chronic feeding with HFD for 8 weeks induced obesogenic phenotype in adult male rats, compared to control animals on the standard chow diet. The HFD rats had a higher caloric intake (Figure 2A) and a 40 g higher body weight gain after 8 weeks as compared to controls (Figure 2B). Moreover, there was a 2-fold increase in pWAT mass in HFD-fed animals compared to controls (Figure 2C). These results were in line with other literature available on diet-induced obesity (DIO) in rodents (14, 25). To assess glycemic status at the time of death, we evaluated glucose and insulin concentrations in plasma over the 24 h cycle. Control animals showed the expected daily rhythm in glucose concentrations in the plasma (26). However, HFD-fed animals showed increased glucose concentrations during the light phase at ZT6 (inactive period) (Figure 2D). The overall high levels of glucose concentration in both conditions could be explained by our choice of euthanasia (60% CO_2_/40% O_2_), as it has been shown previously that CO_2_ causes acidosis which stimulates enzymes of the glycolytic pathway, leading to decreased liver glycogen stores and increased plasma glucose concentrations, both in fed and fasted animals (27, 28). Insulin concentrations were significantly elevated in HFD-fed animals during the dark phase (active period) at ZT18, which could indicate an impaired insulin sensitivity, as glucose concentrations during this period were not elevated, but overall maintained during 24 h (Figure 2E). A similar trend of increased insulin secretion during the dark phase has also been observed in mice on a HFD (29). Evaluation of the NEFA concentrations in plasma showed a significant increase in HFD-fed animals during the light phase (ZT2-ZT10) compared to chow controls (Figure 2F). Together, these data indicate metabolic changes toward obesity in animals fed HFD.

### HFD Disturbs Microglial Circadian Gene Expression

It has been shown previously that microglial cells express clock genes (30, 31). Diets rich in fat and/or sugar are known to alter circadian rhythms of clock gene expression in peripheral tissue (32, 33). To test whether HFD also disturbs daily microglial rhythmicity, we studied expression of genes within the transcriptional feedback loop—circadian locomotor output cycles kaput (*Clock)* and brain and muscle ARNT-Like 1 (*Bmal1)*—the so-called activators and the repressors—period and cryptochrome genes (*Per1, Per2, Cry1*, and *Cry2)*. Additionally, we assessed the expression of two other clock genes—reverse viral erythroblastosis oncogene product alfa (*Reverb*α*)*, a *Clock* and *Bmal1* repressor, and D-box binding protein (*Dbp*), a regulator of peripheral circadian input (34).

**Figure 3:**
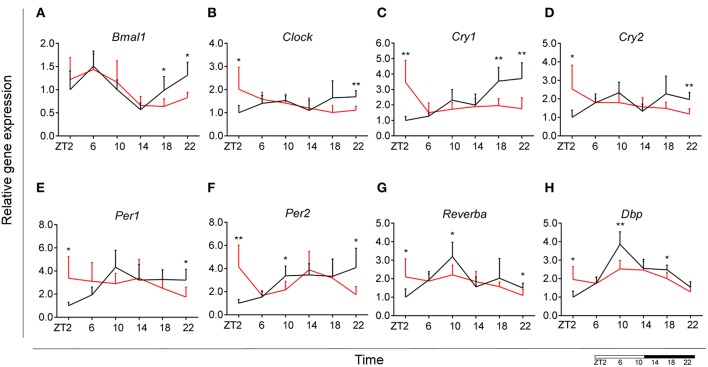
Microglial circadian clock system in HFD-fed and control animals. Relative gene expression of circadian genes *Bmal1* **(A)**, *Clock* **(B)**, *Cry1* **(C)**, *Cry2* **(D)**, *Per1* **(E)**, *Per2* **(F)**, *Reverba* **(G)**, and *Dbp* **(H)** in HFD-fed rats (red) compared to Chow-fed controls (black). Data are presented as means ± SEM. Statistical significance was determined using Two-way ANOVA effects for *Interaction, Diet*, and *Time* (ZT); Student *t*-test is used for diet effect within a separate time point (*p < 0.05; **p < 0.01). Scale (bottom right) represents light (ZT0-12) and dark (ZT12-24) phase.

Control animals fed chow diet showed a clear rhythmic expression for all genes, except *Clock* and *Cry2* (see Table S2). Rhythmicity of *Bmal1, DBP*, and *Reverb*α was not influenced by HFD, although a reduced amplitude was observed for *DBP* and *Reverb*α. There was a gain of rhythm for *Clock* expression. However, *Per1, Per2*, and *Cry1* showed a loss of rhythmic expression during HFD, as evaluated with JTK_Cycle (see Table S2). Moreover, all genes showed a significant *Interaction* effect, as well as difference between HFD and chow-fed animals at the transition period between dark and light phase (ZT22 and/or ZT2) (Figures 3A–H; Table 1). These data point to a clock disturbance, which could lead to irregularity in the expression of other key microglial genes, as it is known that clock genes regulate the expression of 10–20% of all cell genes (34).

**Table 1:** Two-way ANOVA assessment of effect of *Time, Diet*, and *Interaction* in microglia.

**Genes**	**Two-way ANOVA analysis**		
	***p*****-value**		
	***Interaction***	***Time***	***Diet***
**Circadian**			
*Bmal1*	**0.0356**	**<0.0001**	0.3308
*Clock*	**0.0006**	0.4015	0.9599
*Cry1*	**<0.0001**	**0.0002**	0.1484
*Cry2*	**<0.0001**	0.1616	0.7026
*Per1*	**0.0011**	**0.0437**	0.9673
*Per2*	**<0.0001**	**0.0035**	0.9989
*Reverba*	**0.0023**	**<0.0001**	0.5351
*Dbp*	**<0.0001**	**<0.0001**	0.0573
**Inflammatory**			
*Tnfa*	0.1547	**0.0042**	0.1705
*Il1b*	**0.0085**	0.2610	0.4658
*Myd88*	**0.0378**	**0.0022**	**0.0140**
*Ikbkb*	**<0.0001**	**0.0165**	0.7572
*Cd68*	0.0685	**0.0226**	**0.0014**
*Sirt1*	**0.0004**	0.2071	0.4654
**Metabolic**			
*Gls*	**0.0138**	0.4456	0.0888
*Gdh*	**0.0095**	0.8306	0.8648
*Gpx1*	**0.0009**	0.9390	0.8424
*Hk2*	**0.0023**	**0.0274**	**0.0024**
*Glut5*	**0.0058**	0.0935	**0.0025**
*Cd36*	**0.0031**	**0.0004**	0.1116
*Lpl*	0.0554	**0.0064**	**0.0412**
*Ppard*	**0.0034**	0.6143	0.7868
*Fas*	**0.0030**	0.5214	0.6840
**Mitochondrial**			
*Cox4*	**<0.0001**	0.1598	0.8468
*Atp5b*	**0.0020**	**0.0257**	**0.0194**
*Pdk4*	0.2512	**<0.0001**	0.7817
*Fis1*	**0.0001**	0.3438	0.0934
*Drp1*	**<0.0001**	0.2189	0.6340
*Mfn2*	**0.0001**	0.4675	0.1899
*Opa1*	**0.0056**	0.9284	0.6975

*Diet, Time, and Interaction effects were evaluated in microglia for circadian, inflammatory, metabolic, and mitochondrial genes. Statistical significance was determined using Two-way ANOVA effect for Interaction, Diet, and Time (ZT). Data are presented as means ± SEM. Genes are considered rhythmic when p < 0.05 (Bold)*.

### Microglial Time-of-Day Disturbance of Inflammatory Signaling During HFD

**Figure 4:**
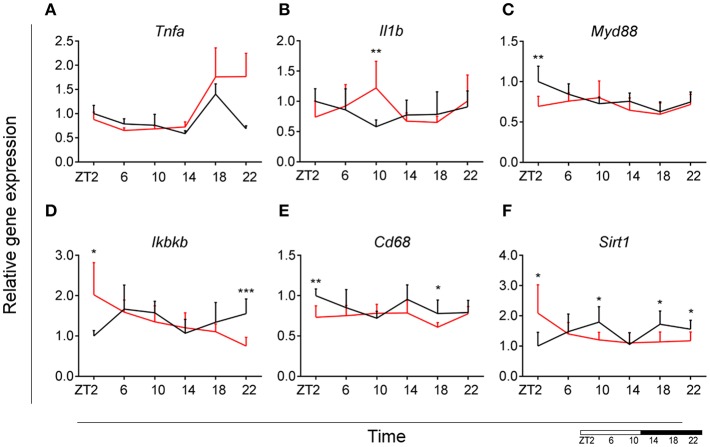
Microglial inflammatory signaling is disturbed in DIO. Relative gene expression of innate immunity genes *Tnfa* **(A)**, *Il1b* **(B)**, *Myd88* **(C)**, *Ikbkb* **(D)** as well as phagocytic indicator gene *Cd68* **(E)** and *Sirt1* **(F)** in HFD-fed rats (red) compared to Chow-fed controls (black) evaluated at six time points, starting at ZT2. Data are presented as means ± SEM. Statistical significance was determined using Two-way ANOVA effect for *Interaction, Diet*, and *Time* (ZT); Student *t*-test is used for diet effect within a separate time point (*p < 0.05; **p < 0.01; ***p < 0.001). Scale (bottom right) represents light (ZT0-12) and dark (ZT12-24) phase.

To evaluate the effect of HFD on daily changes in microglial activation, we assessed the relative gene expression of the main cytokines secreted by microglia—tumor necrosis factor α (*Tnfa*) and interleukin 1β (*Il1b)*. We observed an increased expression of *Tnfa* at the transition between dark and light phase, as well as increased *Il1b* production at the end of the light period for animals fed HFD, pointing to an increased microglial activation in the obesogenic group, compared to controls (Figures 4A,B). However, myeloid differentiation primary response 88 (*Myd88)* gene expression, an adaptor for inflammatory signaling pathways, located downstream of Il1b, showed a decrease at ZT2 in HFD-fed animals (Figure 4C). Therefore, we assessed the expression of inhibitor of nuclear factor kappa B kinase subunit betta (*Ikbkb*) as the protein it encodes phosphorylates the inhibitor in the inhibitor/NFkB complex, leading to activation of nuclear factor kappa-light-chain-enhancer of activated B cells NFkB—a transcriptional activator of key genes involved in cell survival, proliferation and inflammatory response. We observed an inverted daily pattern of *Ikbkb* expression between chow and HFD animals, with higher expression at the beginning of the light phase, but lower expression at the end of the dark phase for HFD-fed animals, compared to chow diet controls (Figure 4D). We also studied gene parameters reflecting the phagocytic capacity of microglia as this is a key function of their immune response in health, as well as different pathologies (35). We evaluated the gene expression of cluster of differentiation 68 (*Cd68*), which encodes for a microglial lysosomal protein, and is a good indicator of phagocytic activity (36). Our results showed an overall steady expression of *Cd68* during the day-night cycle for HFD-fed animals, with a loss of the time-of-day differences, as observed in control animals (Figure 4E). One-Way ANOVA evaluation of the effect of *Time* for each group showed a loss of significance during HFD (see Table S3). Recent studies have shown that Sirtuin 1 (*Sirt1*) deficiency in microglia is associated with increased Il1b production (37). We observed an inverted pattern of expression of *Sirt1* expression in animals fed HFD, compared to controls. Moreover, the significantly lower *Sirt1* expression at ZT10 coincided with an increased expression of *Il1b* at the same time point (Figure 4F). No significant daily rhythmicity was observed for any of the genes, apart from *Myd88* in Chow-fed animals and *Ikbkb* in HFD-fed animals (see Table S2). These data demonstrate that microglial innate immunity is affected in HFD-fed animals, suggesting a disruptive effect of obesogenic diets on the microglial inflammatory response.

### Microglial Glutamate Metabolism Decreases During the Dark Phase During HFD

**Figure 5:**
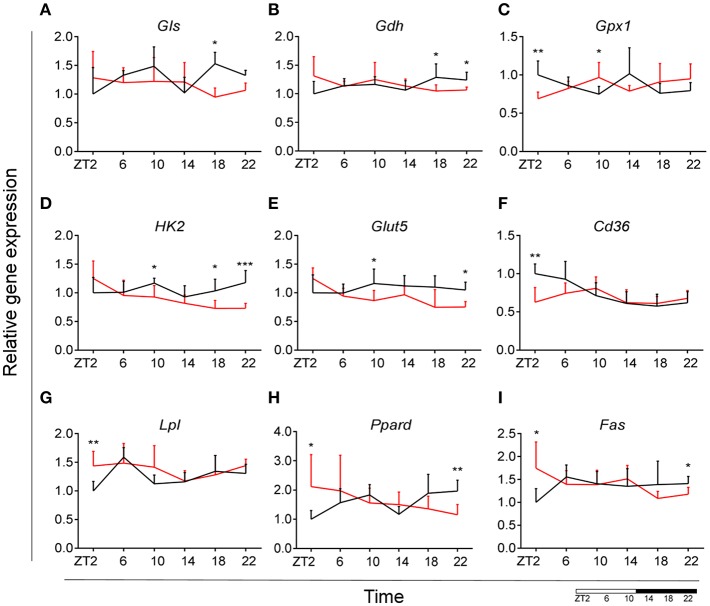
HFD effect on glutamate, glucose and lipid microglial metabolism in rats. Relative gene expression of (top) glutamate substrate utilization genes *Gls* **(A)** and *Gdh* **(B)**, as well as antioxidant enzyme gene *Gpx1* **(C)**; (middle) glucose metabolism genes *Hk2* **(D)** and *Glut5* **(E)**, fatty acid sensing gene *Cd36* **(F)**; (bottom) fatty acid sensing genes *Lpl* **(G)** and *Ppard* **(H)**, as well as fatty acid synthesis gene *Fas* **(I)** in HFD-fed rats (red) compared to Chow-fed controls (black) evaluated at six time points, starting at ZT2. Data are presented as means ± SEM. Statistical significance was determined using Two-way ANOVA effect for *Interaction, Diet*, and *Time* (ZT); Student *t*-test is used for diet effect within a separate time point (*p < 0.05; **p < 0.01; ***p < 0.001). Scale (bottom right) represents light (ZT0-12) and dark (ZT12-24) phase.

Glutamate metabolism is a key component in the biosynthesis of nucleic acids and proteins (38, 39). Microglial cells have been shown to be involved in glutamate uptake under physiological conditions, which can be directly converted to glutathione as a defense response against oxidative stress (40). This mechanism has also been observed under pathological conditions, where it has been shown that microglial cells express glutamate transporters (41). We wanted to assess the state of glutamate substrate utilization in microglial cells under control and obesogenic conditions. We observed that glutaminase (*Gls*)—a key enzyme in the glutamate pathway that converts glutamine to glutamate, showed an effect of *Time* in control animals, which was lost during HFD, with a decrease in expression during the dark phase (ZT18) (Figure 5A) (see Table S3). Similar observations were made for glutamate dehydrogenase 1 (*Gdh1*), a mitochondrial matrix enzyme that converts glutamate to α-ketoglutarate, a key intermediate in the tricarboxylic acid cycle. *Gdh1* expression showed a lower expression during the dark phase for HFD-fed animals (Figure 5B). Moreover, both genes show a significant *Interaction* effect between time and diet (Table 1). These data indicate a decrease in conversion of glutamate during the active state of the animals. Microglial activation leads to production of reactive oxygen species (ROS), therefore self-produced antioxidants could have a protective role in the cells. Expression of glutathione peroxidase 1 (*Gpx1*)—an important antioxidant enzyme, involved in reduction of organic hydroperoxides and hydrogen peroxide by glutathione, showed an inverted pattern of expression during the light phase between both groups (Figure 5C), suggesting a change in this protective mechanism. No significant daily rhythmicity according to JTK_Cycle analysis was observed for any of the genes under control and obesogenic conditions (see Table S2). Together, these data point to an overall decrease of glutamate utilization during the active period of HFD-fed animals.

### Decrease of Microglial Glucose Utilization During the Dark Phase During HFD

It has been shown that glycolysis is crucial for immune cell function (42). Moreover, it has been suggested that upregulation of expression of glycolytic genes leads to M1 polarization in macrophages, known for its proinflammatory function (43). To assess the involvement of glucose metabolism in microglial immune function when rats are fed HFD, we evaluated gene expression of hexokinase 2 (*Hk2*)—the first glycolytic enzyme converting glucose to glucose-6-phosphate. We observed a decrease of *Hk2* expression during the dark phase (ZT18-22) for animals fed HFD, suggesting a decrease in glucose utilization in microglial cells (Figure 5D). Moreover, there was a gain of rhythm for *Hk2* in animals, fed HFD (see Table S2). To investigate this further, we evaluated the expression of glucose transporter type 5 (*Glut5*)—a fructose transporter, which is known to be highly specific for microglial cells (44). We observed a similar trend for *Glut5* in HFD-fed animals, with a steady decreased expression toward the end of the dark phase ZT22 (Figure 5E). Both genes show a significant *Interaction* effect between time and diet (Table 1). Together these data on glutamate and glucose metabolism, suggest that under obesogenic conditions microglial cells switch their substrate utilization to other sources during their active state.

### HFD Leads to an Increase in Lipid Utilization and Sensing in Microglia During the Light Phase

Fatty acid oxidation can contribute 20% of total brain energy production (45). A recent study has shown that microglial cells determine hypothalamic inflammation in response to excess saturated fat intake through a direct and specific sensing mechanism (16). To assess microglial fatty acid (FA) metabolism in DIO, we evaluated genes involved in FA substrate utilization and sensing. Expression of cluster of differentiation 36 (*Cd36*)—a FA translocase responsible for import of FA inside the cell, showed a flattening of the time-of-day differences in animals fed HFD, compared to controls (Figure 5F). Evaluation of daily rhythmicity of *Cd36* gene expression confirms this observation, with a loss of rhythm under obesogenic conditions (see Table S2). This suggests an overall steady import of FA during the day/night cycle under HFD. Previous research from our group has shown that HFD stimulates the expression of microglial lipoprotein lipase (*Lpl*)—a triglyceride hydrolase receptor involved in receptor-mediated lipoprotein uptake, and that lack of LPL impairs microglial immune reactivity (46). Here, we show that this increase of *Lpl* expression takes place during the light phase in animals fed HFD (Figure 5G). These data highlight LPL as a key player in microglial immunometabolism in DIO. Peroxisome proliferator-activated receptors (PPARs) have an important physiological role in lipid sensing and regulation of lipid metabolism during normal healthy conditions, as well in the development of pathologies like obesity and type two diabetes (47). PPAR delta (*Ppard*) is highly expressed by microglia and its activity increases oxidative capacity. Our results showed an inverted pattern of *Ppard* day/night expression in obesogenic animals, with highest expression during ZT2, but lowest at ZT22 (Figure 5H). To assess the effect of HFD-induced obesity on fatty acid synthesis we evaluated gene expression of fatty acid synthase (*Fas*)—a key enzyme catalyzing the synthesis of palmitate from malonyl coenzyme A. *Fas* expression in microglia from HFD-fed animals showed a lower expression at the end of the dark phase and higher expression at the beginning of the light phase, compared to control chow-fed animals (Figure 5I). These data suggest a shift of FA synthesis to the light phase in HFD-fed animals.

Taken together, these data suggest an overall increase in lipid metabolism during the light, i.e., sleep, phase of animals fed HFD. This increase could be partially explained by the higher levels of NEFA in HFD-fed rodents during the light phase (Figure 2F) (48, 49). Moreover, we observed a decrease in glutamate and glucose utilization as shown above. This could suggest a microglial metabolic switch to lipid substrate utilization in HFD-induced obesity.

### HFD Increases Mitochondrial Bioenergetics and Dynamics Gene Expression During the Light Phase

**Figure 6:**
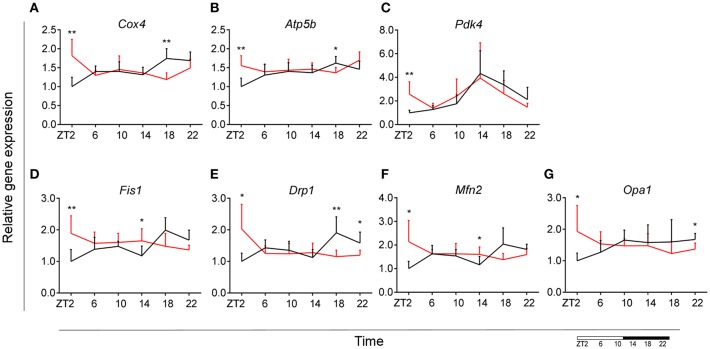
Microglial mitochondria signaling during DIO. Relative gene expression of mitochondria bioenergetics genes *Cox4* **(A)**, *Atp5b* **(B)**, and *Pdk4* **(C)**, as well as mitochondria dynamics gene *Fis1* **(D)**, *Drp1* **(E)**, *Mfn2* **(F)**, and *Opa1* **(G)** in HFD-fed rats (red) compared to Chow-fed controls (black) evaluated at six time points, starting at ZT2. Data are presented as means ± SEM. Statistical significance was determined using Two-way ANOVA effect for *Interaction, Diet*, and *Time* (ZT); Student *t*-test is used for diet effect within a separate time point (*p < 0.05; **p < 0.01). Scale (bottom right) represents light (ZT0-12) and dark (ZT12-24) phase.

To assess whether microglial mitochondria bioenergetics are affected by DIO, we evaluated the gene expression of cytochrome c oxidase subunit 4 (*Cox4*), encoding a terminal enzyme of the mitochondrial respiratory chain that catalyzes the reduction of oxygen to water, and ATP synthase subunit beta (*Atp5b*)—encoding a part of the enzyme, catalyzing ATP synthesis. We observed a decrease in *Cox4* and *Atp5b* expression in animals fed HFD at ZT18 (dark phase), but an increase during the beginning of the light phase (ZT2), suggesting a shift of energy production to the resting state in obese animals (Figures 6A,B). These data are in line with our observation on lipid metabolism; therefore, we selected another mitochondrial target, involved in FA metabolism. Pyruvate dehydrogenase kinase 4 (*Pdk4*) is an enzyme located in the mitochondrial matrix, inhibiting the pyruvate dehydrogenase complex and exerting a regulatory function on substrate utilization by suppressing glycolysis and enhancing FA oxidation. *Pdk4* expression showed the same trend for HFD-fed animals, with an increase at ZT2 (beginning of the light phase) (Figure 6C). This has also been previously observed in heart tissue and soleus muscle of rats fed HFD (49). Moreover, all three genes show a daily rhythm under control conditions, which was lost in HFD-fed animals, suggesting that hypercaloric diet impairs time-of-day mitochondrial bioenergetics in microglial cells (see Table S2).

To test if this trend was also observed in mitochondrial dynamics, as they adjust to mitochondrial demand, we evaluated key genes involved in mitochondrial fusion—mitofusin 2 (*Mfn2*) and optic atrophy 1 (*Opa1*); as well as mitochondrial fission—fission 1 (*Fis1*) and dynamin-related protein 1 (*Drp1*). Results were supportive of changes in the bioenergetics state, with a significant increase of expression for all four genes (*Mfn2, Opa1, Fis1, Drp1*) at ZT2 for HFD-fed animals (Figures 6D–G). Two-way ANOVA test showed a significant *Interaction* effect for all four genes (Table 1).

Taken together these data suggest an increased energy production in microglia of DIO animals during the light phase, which could be explained by an increased demand to sustain the increase in lipid metabolism. Another recent study indeed showed that mitochondrial fission is elevated as a consequence of high-fat concentrated diets (50). This indicates that mitochondrial dynamics adapt to changes in the bioenergetics state in response to nutritional status.

### The Effect of HFD-Induced Obesity on Blood Monocyte Immunometabolism Is Less Robust Than on Brain Microglial Cells

Following our observations in microglia, we were interested if the same effects could be seen in monocytes—peripheral myeloid cells. Originating from hematopoietic stem cells in the bone marrow, monocytes circulate in the blood and migrate to other tissue where they differentiate into tissue resident macrophages. It is known that under obesogenic conditions, circulating monocytes could infiltrate adipose tissue, leading to macrophage activation and increasing proinflammatory activity (51–53).

**Figure 7:**
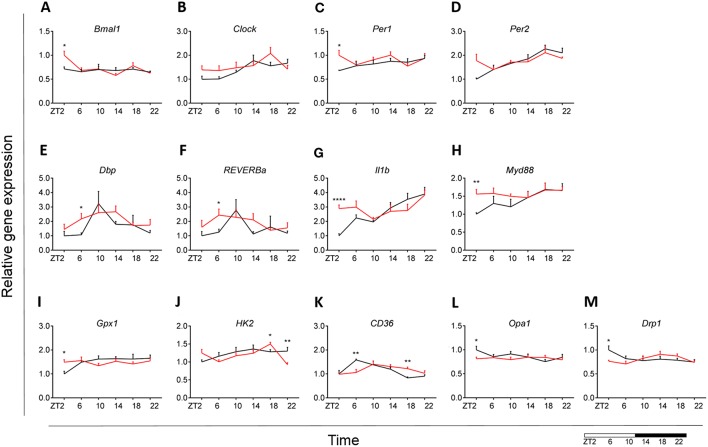
Monocyte immunometabolism in DIO. Relative gene expression of circadian genes *Bmal1* **(A)**, *Clock* **(B)**, *Per1* **(C)**, *Per2* **(D)**, *Dbp* **(E)**, and *Reverba* **(F)**; immune genes *Il1b* **(G)** and *Myd88* **(H)**, antioxidant enzyme gene *Gpx1* **(I)**, glycolysis gene *Hk2* **(J)**, fatty acid sensing gene *Cd36* **(K)**, and mitochondria dynamic genes *Opa1* **(L)** and *Drp1* **(M)** in HFD-fed rats (red) compared to Chow-fed controls (black) evaluated at six time points, starting at ZT2. Data are presented as means ± SEM. Statistical significance was determined using Two-way ANOVA effect for *Interaction, Diet*, and *Time* (ZT); Student *t*-test is used for diet effect within a separate time point (*p < 0.05; **p < 0.01; ****p < 0.0001). Scale (bottom right) represents light (ZT0-12) and dark (ZT12-24) phase.

Our results indicated an overall loss of daily rhythmicity of circadian gene expression, with *Clock, Per2*, and *Dbp* showing daily rhythmicity in control animals, which was only maintained for *Per2* gene expression under obesogenic conditions (see Table S2). *Bmal1* and *Per1* showed a significant increase in expression at the beginning of the light phase (ZT2) in HFD-fed animals compared to control chow (Figures 7A,C). Gene expression of *Reverba* and *Dbp* in monocytes showed a higher expression at ZT6 in HFD-fed animals (Figures 7E,F). There was no difference in *Clock*, *Per2, Cry1* and *Cry2* gene expression between both conditions (Figures 7B,D) (see Figures S1A,B). Moreover, One-Way ANOVA analysis showed lack of *Time* effect for all circadian genes during HFD (see Table S3).

We did not find any difference in monocyte immune response between both groups for *Tnfa, Ikbkb, Cd68*, and *Sirt1* gene expression (see Figures S1C–F). However, we did observe a daily rhythm in *Tnfa* and *Cd68* in control animals, as well as gain of rhythm for *Sirt1* gene expression in HFD-fed animals (see Table S2). There was an increase in *Il1b* expression at ZT2 for the HFD group (Figure 7G). *Il1b* showed daily rhythmicity under control conditions, which was maintained under obesogenic conditions with a shift in acrophase of 6 h (see Table S2). Il1b-induced inflammation has been shown to be indirectly involved in insulin resistance in type 2 diabetes (54, 55). Thus, these data could indicate a reduction in insulin sensitivity. Moreover, we observed an increased expression of *Myd88* at ZT2 for HFD-fed animals (Figure 7H).

No differences between obese and control animals were found for representative genes of the glutamate pathway *Gls* and *Gdh* (see Figures S1G,H). However, there was a gain of daily rhythm for *Gls* gene expression in HFD-fed animals (see Table S2). We found an increase in *Gpx1* expression at ZT2 for HFD group with an overall stable day/night expression, suggesting a mechanism of constant anti-oxidant production (Figure 7I). This observation was supported by a loss of daily rhythmicity under obesogenic conditions (see Table S2). Expression of the glucose metabolic gene *Hk2* was decreased at ZT22 in HFD-fed animals, similar to what was observed in microglia (Figure 7J). We observed no difference in FA metabolism and sensing genes *Fas* and *Ppard* (see Figures S1I,J), apart from *Cd36* expression (Figure 7K). *Cd36* expression showed a strong daily rhythm under control conditions, which was significant also in HFD-fed animals with an acrophase shift of 6 h (see Table S2). The expression of the FA translocase in monocytes has also been shown to be associated with insulin resistance, supporting our observation for *Il1b* expression (56). *Lpl* evaluation showed low expression (data not shown).

We observed no difference in mitochondrial bioenergetics gene expression between both dietary groups for *Atp5b, Atp5g*, and *Cox4* (see Figures S1K–M). Mitochondria dynamics gene expression was affected only at ZT2 for *Opa1* and *Drp1* expression (Figures 7L,M), with no difference in *Mfn2* expression (see Figure S1N) and low expression of *Fis1* (data not shown). Interestingly, HFD led to a decrease in mitochondrial bioenergetics gene expression in monocytes at the start of the inactive period, opposite to the increase we observed in microglia under obesogenic conditions. We found no daily rhythm for any of the mitochondria genes, both under control and obesogenic conditions (see Table S2). One-Way ANOVA analysis showed lack of *Time* effect for all genes both during control and HFD (see Table S3). Two-way ANOVA analysis data is shown the in Supplementary Material (see Table S4).

Overall, these data suggest a small effect of the obesogenic diet on monocyte immunometabolism, suggesting that HFD specifically affects microglial immunometabolism.

## Discussion

It is well-known now that a hypercaloric environment is a potent inducer of microglial activation, which ultimately leads to chronic neuroinflammation (14–17). However, the daily rhythm of microglial innate immune function is poorly known, both in obesity and health. The purpose of this study was to evaluate the effect of an obesogenic diet on daily changes in microglial immunometabolism. Our data showed a disturbance of the microglial interaction between metabolism and immunity during DIO. We report that HFD-induced obesity leads to loss of daily rhythm of circadian genes and impaired microglial immunometabolic functions primarily at the transition period between dark and light phase (ZT22-ZT2).

To evaluate the effect of DIO on daily rhythms in microglial function and activity, we studied the microglial expression of major circadian and immune genes. Under normal conditions, microglia circadian genes were expressed in a rhythmic manner, which is disturbed by HFD, mainly due to a loss of its rhythmicity. Comparable changes have also been observed in different peripheral tissues like liver, brown adipose tissue and skeletal muscle in animals on an obesogenic diet (57–59). However, to our knowledge, we are the first to report an effect of HFD on expression rhythms of microglial clock genes. The presently reported difference in time-of-day expression of microglial cytokine genes, is in line with our previous results (18). Fonken et al. have shown previously that *Il1b* and *Tnfa* gene expression have a peak during the middle of the day, contrary to our observations (31). Possible explanation to this contradiction is the heterogeneous transcriptional identities of microglia, specific for each brain region, in this case hippocampal vs. cortical microglia (60).

Microglial cells are known to exhibit bioenergetics shifts in energy substrate, for example during aging (61). Such a shift in substrate utilization is known to have an effect on the activation status of immune cells (42, 62). We studied microglial substrate utilization, focusing on glutamate, glucose and FA metabolism and observed a difference between control and HFD-fed animals, particularly during the transition period from the dark to light phase. Key players in the glutamate pathway have been shown to be involved in macrophage immune function, *e.g.*, glutamine availability was shown to modulate macrophage phagocytic capacity, while α-ketoglutarate, generated through glutaminolysis, is crucial in eliciting an anti-inflammatory phenotype in macrophages (63, 64). We report a decrease in microglial glutamate utilization in the active period of HFD-fed animals as seen in glutamine conversion to glutamate and glutamate conversion to α-ketoglutarate. Additionally, a similar change was observed for glucose metabolism with decreased glucose utilization in the active period of HFD-fed animals. However, we observed an increase in FA sensing and synthesis at the beginning of the light period under obesogenic conditions, suggesting a shift to FA utilization during the sleep phase of the animal. It has been shown that FA treatment of BV2 cells (a microglial cell line) is a potent inducer of cytokine production via TLR4 signaling, thus leading to low-grade inflammation even in the absence of immune challenge (65). This FA metabolism increase could be a possible explanation for our previously observed constant day/night activation of hypothalamic microglia under HFD (66). Additionally, we know that immune cell activation requires higher energy production. We here show that microglial mitochondrial function in DIO is increased during the inactive period, suggesting an increase in ATP production, which could be explained by the increased FA metabolism demand. These data support the view that mitochondrial function adapts to nutritional status (50).

To investigate whether the observed effect of HFD on immunometabolism is restricted to microglial cells, we also studied monocyte immunometabolism in obesity. We report small or no effect of the hypercaloric diet on monocyte immunometabolic function, which suggests a microglia-specific functional disturbance in HFD-induced obesity. Taken together, our data suggest that microglial innate immunity is highly dependent on metabolic changes, as well as the time of day. Microglial cells are highly active cells, with a high energy demand, which is achieved by a strictly regulated cellular metabolism. A robust switch of substrate utilization is a suitable mechanism, in response to the high demands of immune defense.

The data currently presented suggest a deleterious effect of an obesogenic diet on microglial function by inducing chronic activation. It has been shown that chronic microglial activation has a negative impact on neuronal function and could play a role in obesity-associated cognitive decline (16, 67). Our data point out to the importance of microglial integrity and the negative impact of chronic exposure to a hypercaloric environment on cortical microglial function, which could ultimately lead to cognitive impairment. Previously we observed that obesity induces microglial activation in close proximity to the anorexigenic proopiomelanocortin (POMC) neurons located in the arcuate nucleus of the hypothalamus (18). Moreover, chronic HFD feeding leads to POMC neuronal loss, which would lead to further progression of obesity (66). It is possible that the current observation on cortical microglia could be translated to the hypothalamus, which would give insight in the mechanisms behind this neuronal loss.

Finally, three issues need to be addressed: firstly, we observed a clear effect of HFD on microglial immunometabolism, leading to an increase in expression of many of the presented genes around the end of the dark period, i.e., ZT22/ZT2. In order to check whether or not a higher food intake at the end of the dark period in the HFD-fed group could be responsible for these changes, we re-analyzed the food intake data from metabolic cage experiments from a separate cohort of rats fed a similar HFD (68). With respect to consumed grams, no difference in timing of food intake was found between control and obesogenic diet (see Figure S2). However, with respect to consumed calories, the obesogenic diet group showed a larger increase of kcal intake at the beginning and the end of the dark period, although only significant for the beginning of the dark period, suggesting that higher energy consumption (but not higher food intake) may be partially responsible for the differences in gene expression between the HFD and control group at the end of the dark period (see Figure S2). Secondly, we cannot distinguish between the effect of obesity and the hypercaloric diet itself. However, a hypercaloric diet can induce microglial activation in the hypothalamus after 1 day, prior to any changes in body weight, pointing to an effect of diet rather than obesity itself (69). Thirdly, the data presented only show the transcriptional state of selected target genes, representative of the different functions investigated. Future studies should be aimed at a further understanding of activity changes in each of the represented pathways.

## Conclusions

An obesogenic diet affects microglial immunometabolism in a time-of-day specific manner. The aim of this study was to increase the knowledge of microglial cell function in obesity in general and its daily rhythms in specific. To our knowledge, we are the first to point out (loss of) time-of-day differences for microglial cells during HFD. Our data are supportive of the ongoing research, focused on the interaction between immune cells and metabolism. Further studies should focus on addressing the time-of-day differences in microglial function, as more detailed knowledge of microglial immunometabolism could lead to a better understanding of the neuroinflammatory process taking place in the CNS under chronic hypercaloric environment.

## Data Availability

All data generated or analyzed during this study are included in this published article (and its Supplementary Information Files).

## Ethics Statement

All studies were approved by the Animal Ethics Committee of the Royal Dutch Academy of Arts and Sciences (KNAW, Amsterdam) and performed according to the guidelines on animal experimentation of the Netherlands Institute for Neuroscience (NIN, Amsterdam).

## Author Contributions

IM performed the animal experiments, microglia isolation, RNA extraction, cDNA synthesis, qPCR experiments, glucose and NEFA measurements in plasma, and constructed the manuscript. MK and XW helped with the animal experiments and monocyte isolation. NK performed the monocyte isolation and helped with the animal experiments. DS performed the time-of-day food intake data and helped with data analysis. SW helped with qPCR experiments. PG helped with data statistical analysis. AH supervised the measuring of the insulin plasma concentration. EF, SF, and AK provided intellectual input and drafted the manuscript. CY designed the study, supervised the experiments, interpreted the findings, and drafted the manuscript. All authors have read and approved the final manuscript.

### Conflict of Interest Statement

The authors declare that the research was conducted in the absence of any commercial or financial relationships that could be construed as a potential conflict of interest.

## Abbreviations

(Abbreviations in italic represent target genes tested) *Atp5b*: ATP synthase subunit b
*Atp5g*: ATP synthase subunit g
*bactin*: Beta-actin
*Bmal1*: Brain and muscle ARNT-Like 1
*Cd36*: Cluster of differentiation 36
*Cd68*: Cluster of differentiation 68
*Clock*: Circadian locomotor output cycles kaput
*Cox4*: Cytochrome C oxidase subunit 4
*Cry1*: Cryptochrome 1
*Cry2*: Cryptochrome 2
*Dbp*: D-box binding protein
DIO: Diet-induced obesity
*Drp1*: Dynamin-related protein 1
EDTA: Ethylenediaminetetraacetic acid
FA: Fatty acid
*Fas*: Fatty acid synthesis
*Fis1*: Fission 1
*Gdh*: Glutamate dehydrogenase
*Gls*: Glutaminase
*Glut5*: Glucose transporter type 5
*Gpx1*: Glutathione peroxidase 1
HFD: High-carbohydrate-high-fat diet
*Hk2*: Hexokinase 2
*Hprt*: Hypoxanthine phosphoribosyltransferase 1
*Ikbkb*: Inhibitor of nuclear factor kappa B kinase subunit betta
*Il1b*: Interleukin 1 betta
*Lpl1*: Lipoprotein lipase 1
*Mfn2*: Mitofusin 2
*Myd88*: Myeloid differentiation primary response 88
NEFA: Non-esterified fatty acids
*Opa1*: Optic atrophy 1
*Pdk4*: Pyruvate dehydrogenase kinase 4
*Per1*: Period 1
*Per2*: Period 2
POMC: Proopiomelanocortin
*Ppard*: Peroxisome proliferator activated receptor delta
pWAT: Perirenal white adipose tissue
*Reverba*: Reverse viral erythroblastosis oncogene product alfa
RIA: Radioimmunoassay
ROS: Reactive oxygen species
SEM: Standard error of the mean
*Sirt1*: Sirtuin 1
*Tnfa*: Tumor necrosis factor alfa
ZT: Zeitgeber Time.
