# Supplementary material for: Diet-Induced Obesity Disturbs Microglial Immunometabolism in a Time-of-Day Manner
Source: Front Endocrinol (Lausanne). 2019 Jun 26;10:424. doi: 10.3389/fendo.2019.00424 (PMC6611391; doi:10.3389/fendo.2019.00424)
Supplement: Supplementary file 1 [file Table_1.docx]

| **Genes** | **Primer sequence - Forward** | **Primer sequence - Reverse** |
| --- | --- | --- |
| **Circadian** |  |  |
| *Bmal1* | CCGATGACGAACTGAAACACCT | TGCAGTGTCCGAGGAAGATAGC |
| *Clock* | CGATCACAGCCCAACTCCTT | TTGCAGCTTGAGACATCGCT |
| *Cry1* | AAGTCATCGTGCGCATTTCA | TCATCATGGTCGTCGGACAGA |
| *Cry2* | TGGATAAGCACTTGGAACGGAA | TGTACAAGTCCCACAGGCGGTA |
| *Per1* | CGCACTTCGGGAGCTCAAACTTC | GTCCATGGCACAGGGCTCACC |
| *Per2* | CACCCTGAAAAGAAAGTGCGA | CAACGCCAAGGAGCTCAAGT |
| *Reverba* | ACAGCTGACACCACCCAGATC | CATGGGCATAGGTGAAGATTTCT |
| *Dbp* | CCTTTGAACCTGATCCGGCT | TGCCTTCTTCATGATTGGCTG |
| **Inflammatory** |  |  |
| *Tnfa* | AACACACGAGACGCTGAAGT | TCCAGTGAGTTCCGAAAGCC |
| *Il1b* | TGTGATGAAAGACGGCACAC | CTTCTTCTTTGGGTATTGTTTGG |
| *Myd88* | TCGACGCCTTCATCTGCTAC | CCATGCGACGACACCTTTTC |
| *Ikbkb* | GCAGAACTTGGCACCCAATG | GAGCCGATGCTATGTCACTCA |
| *Cd68* | TGTTCAGCTCCAAGCCCAAA | GCTCTGATGTCGGTCCTGTTT |
| *Sirt1* | TGTTTCCTGTGGGATACCTGA | TGAAGAATGGTCTTGGGTCTTT |
| **Metabolic** |  |  |
| *Gls* | TACGACTCCAGAACAGCCCT | TTATTCCACCTGTCCTTGGGG |
| *Gdh* | CCTGCAAGGGAGGTATCCG | CCACAGCGCACTTGTATGTC |
| *Gpx1* (microglia)* | CAAGTATGTCCGACCCGGTG | CTCACCATTCACCTCGCACT |
| *Gpx1* (monocytes)* | CCGGGACTACACCGAAATGA | CGGGTCGGACATACTTGAGG |
| *Hk2* | GGTGAGCCATCGTGGTTAAG | CTTCCGGAACCGCCTAGAAA |
| *Glut5* | CTTATTGCCCAGGTGTTCGG | GGCAGAAGGGCAACAGGATA |
| *Cd36* | ACAGTTTTGGATCTTTGACGTG | CCTTGGCTAAATAACGAACTCTG |
| *Lpl* | CAAAACAACCAGGCCTTCGA | AGCAATTCCCCGATGTCCA |
| *Ppard* | CTCCTGCTCACTGACAGATG | TCTCCTCCTGTGGCTGTTC |
| *Fas* | CTTGGGTGCCGATTACAACC | GCCCTCCCGTACACTCACTC |
| **Mitochondrial** |  |  |
| *Cox4* | TGGGAGTGTTGTGAAGAGTGA | GCAGTGAAGCCGATGAAGAAC |
| *Atp5b* | CGGGTAGCTCTGACTGGTCT | AACTCAGCAATAGCACGGGA |
| *Atp5g* | GGAGATAACGGCCAATGGGAG | CACAGGCCTGATTAGACCCC |
| *Pdk4* | TGGTTTTGGTTACGGCTTGC | TGCCAGTTTCTCCTTCGACA |
| *Fis1* | GGTTGCGTGGTAAGGGATGA | CTGTAACAGTCCCCGCACAT |
| *Drp1* | ACAACAGGAGAAGAAAATGGAGTTG | CGTTGGGCGAGAAAACCTTG |
| *Mfn2* | CTCAGGAGCAGCGGGTTTATTGT | TGTCGAGGGACCAGCATGTCTAT |
| *Opa1* | AAGGCATCCACCACAGGAAG | CCTCGTGGGAATATTCGTGCT |
| **Housekeeping** |  |  |
| *Hprt* (origin: *Mus musculus)* | GCAGTACAGCCCCAAAATGG | AACAAAGTCTGGCCTGTATCCAA |
| *bactin* | ACAACCTTCTTGCAGCTCCTC | CTGACCCATACCCACCATCAC |

***Table S1. Primer sequences of target genes***

*Two different primers for *Gpx1* were used for monocyte and microglial cells as the primers showed tissue specificity and a single primer, suitable for both cell types was not found during optimization of the technique.
