# Supplementary material for: Diet-Induced Obesity Disturbs Microglial Immunometabolism in a Time-of-Day Manner
Source: Front Endocrinol (Lausanne). 2019 Jun 26;10:424. doi: 10.3389/fendo.2019.00424 (PMC6611391; doi:10.3389/fendo.2019.00424)
Supplement: Supplementary file 2 [file Table_2.docx]

***Table S2. Daily rhythmicity analysis***

**Table S2. JTK_Cycle analysis of daily rhythmicity.** Diet and Time effect on daily rhythms in microglia and monocytes for circadian, inflammatory, metabolic and mitochondrial genes. Data was analyzed with JTK_Cycle software and p-values were obtained by fitting the data on a curve with fixed 24h period. The acrophase is given for rhythmic genes (in ZT). Genes are considered rhythmic when p<0.05 (**Bold**). N/A = not applicable (gene has low or no expression); NR = not rhythmic.

| **Genes** | **JTK_Cycle analysis for microglia** | | | | **JTK_Cycle analysis for monocytes** | | | |
| --- | --- | --- | --- | --- | --- | --- | --- | --- |
|  | **Chow** | | **HFD** | | **Chow** | | **HFD** | |
|  | p-value | acrophase | p-value | acrophase | p-value | acrophase | p-value | acrophase |
| **Circadian** |  |  |  |  |  |  |  |  |
| *Bmal1* | **0.003** | 2 | **<0.0001** | 4 | 0.09 | NR | 1 | NR |
| *Clock* | 1 | NR | **0.002** | 4 | **0.001** | 16 | 0.22 | NR |
| *Cry1* | **0.003** | 16 | 1 | NR | 1 | NR | 0.25 | NR |
| *Cry2* | 1 | NR | 0.19 | NR | 1 | NR | 1 | NR |
| *Per1* | **0.001** | 14 | 0.63 | NR | 1 | NR | 0.22 | NR |
| *Per2* | **0.003** | 14 | 0.53 | NR | **0.0002** | 16 | **0.008** | 18 |
| *Reverba* | **0.009** | 10 | **0.002** | 8 | 0.56 | NR | 0.25 | NR |
| *Dbp* | **<0.0001** | 12 | **0.0001** | 10 | **0.015** | 12 | 0.07 | NR |
| **Inflammatory** |  |  |  |  |  |  |  |  |
| *Tnfa* | 1 | NR | 0.06 | NR | **0.02** | 20 | 0.1 | NR |
| *Il1b* | 0.07 | NR | 0.26 | NR | **0.002** | 16 | **0.04** | 22 |
| *Myd88* | **0.01** | 4 | 1 | NR | 0.14 | NR | 1 | NR |
| *Ikbkb* | 1 | NR | **0.01** | 6 | 1 | NR | 0.07 | NR |
| *Cd68* | 1 | NR | 0.88 | NR | **<0.0001** | 12 | 0.37 | NR |
| *Sirt1* | 1 | NR | 0.17 | NR | 1 | NR | **0.02** | 18 |
| **Metabolic** |  |  |  |  |  |  |  |  |
| *Gls* | 1 | NR | 0.42 | NR | 1 | NR | **0.001** | 18 |
| *Gdh* | 1 | NR | 0.39 | NR | 1 | NR | 0.44 | NR |
| *Gpx1* | 1 | NR | 1 | NR | **0.04** | 14 | 1 | NR |
| *Hk2* | 1 | NR | **0.01** | 4 | 0.28 | NR | 0.08 | NR |
| *Glut5* | 1 | NR | 0.2 | NR | N/A | N/A | N/A | N/A |
| *Cd36* | **<0.0001** | 4 | 0.28 | NR | **<0.0001** | 6 | **0.007** | 12 |
| *Lpl* | 1 | NR | 0.19 | NR | N/A | N/A | N/A | N/A |
| *Ppard* | 1 | NR | 1 | NR | 1 | NR | 0.63 | NR |
| *Fas* | 1 | NR | 0.25 | NR | 0.25 | NR | 0.15 | NR |
| **Mitochondrial** |  |  |  |  |  |  |  |  |
| *Cox4* | **0.049** | 18 | 0.13 | NR | 1 | NR | 1 | NR |
| *Atp5b* | **0.01** | 16 | 0.56 | NR | 0.26 | NR | 0.42 | NR |
| *Atp5g* | N/A | N/A | N/A | N/A | 1 | NR | 1 | NR |
| *Pdk4* | **<0.0001** | 14 | 0.53 | NR | N/A | N/A | N/A | N/A |
| *Fis1* | 0.07 | NR | 0.79 | NR | N/A | N/A | N/A | N/A |
| *Drp1* | 1 | NR | 0.56 | NR | 0.75 | NR | 0.09 | NR |
| *Mfn2* | 1 | NR | 0.71 | NR | 1 | NR | 1 | NR |
| *Opa1* | 0.12 | NR | 0.42 | NR | 0.53 | NR | 1 | NR |
