# Supplementary material for: Diet-Induced Obesity Disturbs Microglial Immunometabolism in a Time-of-Day Manner
Source: Front Endocrinol (Lausanne). 2019 Jun 26;10:424. doi: 10.3389/fendo.2019.00424 (PMC6611391; doi:10.3389/fendo.2019.00424)
Supplement: Supplementary file 3 [file Table_3.docx]

***Table S3. One-way ANOVA analysis of effect of time in separate feeding groups***

**Table S3. One-way ANOVA assessment of effect of *Time*.** *Time* effect evaluation in separate feeding groups. Statistical significance was determined using One-way ANOVA effect *Time* (ZT). Genes are considered rhythmic when p<0.05 (**Bold**). N/A = not applicable (gene has low or no expression).

| **Genes** | **One-way ANOVA analysis of effect of *Time* for microglia** | | **One-way ANOVA analysis of effect of *Time* for monocytes** | |
| --- | --- | --- | --- | --- |
|  | **Chow** | **HFD** | **Chow** | **HFD** |
|  | p-value | | p-value | |
| **Circadian** |  |  |  |  |
| *Bmal1* | **0.0001** | **0.0004** | **0.0002** | 0.9491 |
| *Clock* | **0.0415** | **0.0148** | **0.0019** | 0.1479 |
| *Cry1* | **<0.0001** | **0.0018** | 0.5351 | 0.3658 |
| *Cry2* | **0.0012** | **0.0202** | 0.4521 | 0.6039 |
| *Per1* | **<0.0001** | 0.2899 | 0.2289 | 0.1508 |
| *Per2* | **0.0001** | **0.0047** | **<0.0001** | 0.1125 |
| *Reverba* | **<0.0001** | **0.0228** | 0.0702 | 0.3788 |
| *Dbp* | **<0.0001** | **0.0004** | **0.0129** | 0.2177 |
| **Inflammatory** |  |  |  |  |
| *Tnfa* | **0.0088** | **0.0387** | **0.0007** | 0.0554 |
| *Il1b* | 0.1805 | **0.0282** | **<0.0001** | 0.0680 |
| *Myd88* | **0.0008** | 0.1974 | 0.0613 | 0.8670 |
| *Ikbkb* | **0.0254** | **0.0005** | 0.5558 | 0.0616 |
| *Cd68* | **0.0312** | 0.1253 | **<0.0001** | 0.4689 |
| *Sirt1* | **0.0204** | **0.0111** | 0.7105 | 0.0823 |
| **Metabolic** |  |  |  |  |
| *Gls* | **0.0142** | 0.4831 | 0.5431 | **0.0026** |
| *Gdh* | 0.0724 | 0.1871 | 0.7926 | 0.2481 |
| *Gpx1* | 0.0600 | **0.0418** | **0.0089** | 0.7746 |
| *Hk2* | 0.2235 | **0.0010** | 0.1326 | **0.0004** |
| *Glut5* | 0.7090 | **0.0007** | N/A | N/A |
| *Cd36* | **0.0003** | 0.1386 | **<0.0001** | **0.0272** |
| *Lpl* | **0.0001** | 0.4000 | N/A | N/A |
| *Ppard* | **0.0010** | 0.2455 | **0.0056** | 0.3960 |
| *Fas* | 0.1286 | **0.0237** | 0.1446 | 0.2566 |
| **Mitochondrial** |  |  |  |  |
| *Cox4* | **<0.0001** | **0.0077** | 0.5976 | 0.3726 |
| *Atp5b* | **0.0018** | 0.1155 | 0.1180 | 0.3308 |
| *Atp5g* | N/A | N/A | 0.4628 | 0.9706 |
| *Pdk4* | **<0.0001** | 0.0733 | N/A | N/A |
| *Fis1* | **0.0005** | 0.2790 | N/A | N/A |
| *Drp1* | **0.0010** | **0.0050** | 0.1263 | 0.0520 |
| *Mfn2* | **0.0008** | 0.1415 | 0.7031 | 0.6176 |
| *Opa1* | 0.0722 | 0.1677 | 0.1210 | 0.9114 |
