# Supplementary material for: Diet-Induced Obesity Disturbs Microglial Immunometabolism in a Time-of-Day Manner
Source: Front Endocrinol (Lausanne). 2019 Jun 26;10:424. doi: 10.3389/fendo.2019.00424 (PMC6611391; doi:10.3389/fendo.2019.00424)
Supplement: Supplementary file 4 [file Table_4.docx]

***Table S4. Two-way ANOVA analysis of monocytes***

**Table S4. Two-way ANOVA assessment of effect of *Time, Diet* and *Interaction* in monocytes.** Statistical significance was determined using Two-way ANOVA effect for *Interaction*, *Diet* and *Time* (ZT). Data are presented as means ± SEM. Genes are considered rhythmic when p<0.05 (**Bold**).

| **Genes** | **Two-way ANOVA analysis** | | |  | **Genes** | **Two-way ANOVA analysis** | | |
| --- | --- | --- | --- | --- | --- | --- | --- | --- |
|  | p-value | | |  |  | p-value | | |
|  | *Interaction* | *Time* | *Diet* |  |  | *Interaction* | *Time* | *Diet* |
| **Circadian** |  |  |  |  | **Metabolic** |  |  |  |
| *Bmal1* | **0.0401** | **0.0024** | 0.1879 |  | *Gls* | 0.6480 | **0.0044** | 0.8767 |
| *Clock* | 0.1578 | **0.0025** | 0.1114 |  | *Gdh* | 0.5924 | 0.3948 | 0.3775 |
| *Cry1* | 0.5488 | 0.3290 | 0.6740 |  | *Gpx1* | **0.0414** | 0.0767 | 0.7417 |
| *Cry2* | 0.7040 | 0.3451 | 0.3935 |  | *Hk2* | **0.0062** | **0.0060** | 0.3444 |
| *Per1* | 0.1331 | 0.2338 | 0.0660 |  | *Cd36* | **0.0012** | **<0.0001** | 0.7674 |
| *Per2* | **0.0226** | **<0.0001** | 0.5676 |  | *Ppard* | 0.4536 | **0.0024** | 0.2261 |
| *Reverba* | 0.3139 | 0.0579 | 0.1016 |  | *Fas* | 0.9708 | **0.0178** | 0.1265 |
| *Dbp* | 0.3748 | **0.0027** | 0.1182 |  |  |  |  |  |
|  |  |  |  |  |  |  |  |  |
| **Inflammatory** |  |  |  |  | **Mitochondrial** |  |  |  |
| *Tnfa* | 0.5031 | **<0.0001** | 0.3107 |  | *Cox4* | 0.7866 | 0.2362 | 0.5052 |
| *Il1b* | **0.0051** | **<0.0001** | 0.1422 |  | *Atp5b* | 0.8743 | **0.0261** | 0.4652 |
| *Myd88* | 0.3699 | 0.0780 | **0.0452** |  | *Atp5g* | 0.8766 | 0.7251 | 0.8313 |
| *Ikbkb* | 0.3379 | 0.1678 | 0.2697 |  | *Drp1* | **0.0392** | 0.1454 | 0.5702 |
| *Cd68* | 0.0681 | **0.0001** | 0.3710 |  | *Mfn2* | 0.8240 | 0.4955 | 0.2219 |
| *Sirt1* | 0.2185 | 0.3303 | 0.2993 |  | *Opa1* | 0.1594 | 0.4131 | 0.1118 |
