# Supplementary material for: Diet-Induced Obesity Disturbs Microglial Immunometabolism in a Time-of-Day Manner
Source: Front Endocrinol (Lausanne). 2019 Jun 26;10:424. doi: 10.3389/fendo.2019.00424 (PMC6611391; doi:10.3389/fendo.2019.00424)
Supplement: Supplementary file 5 [file Data_Sheet_1.docx]

***Figure S1. Additional monocyte data***

***
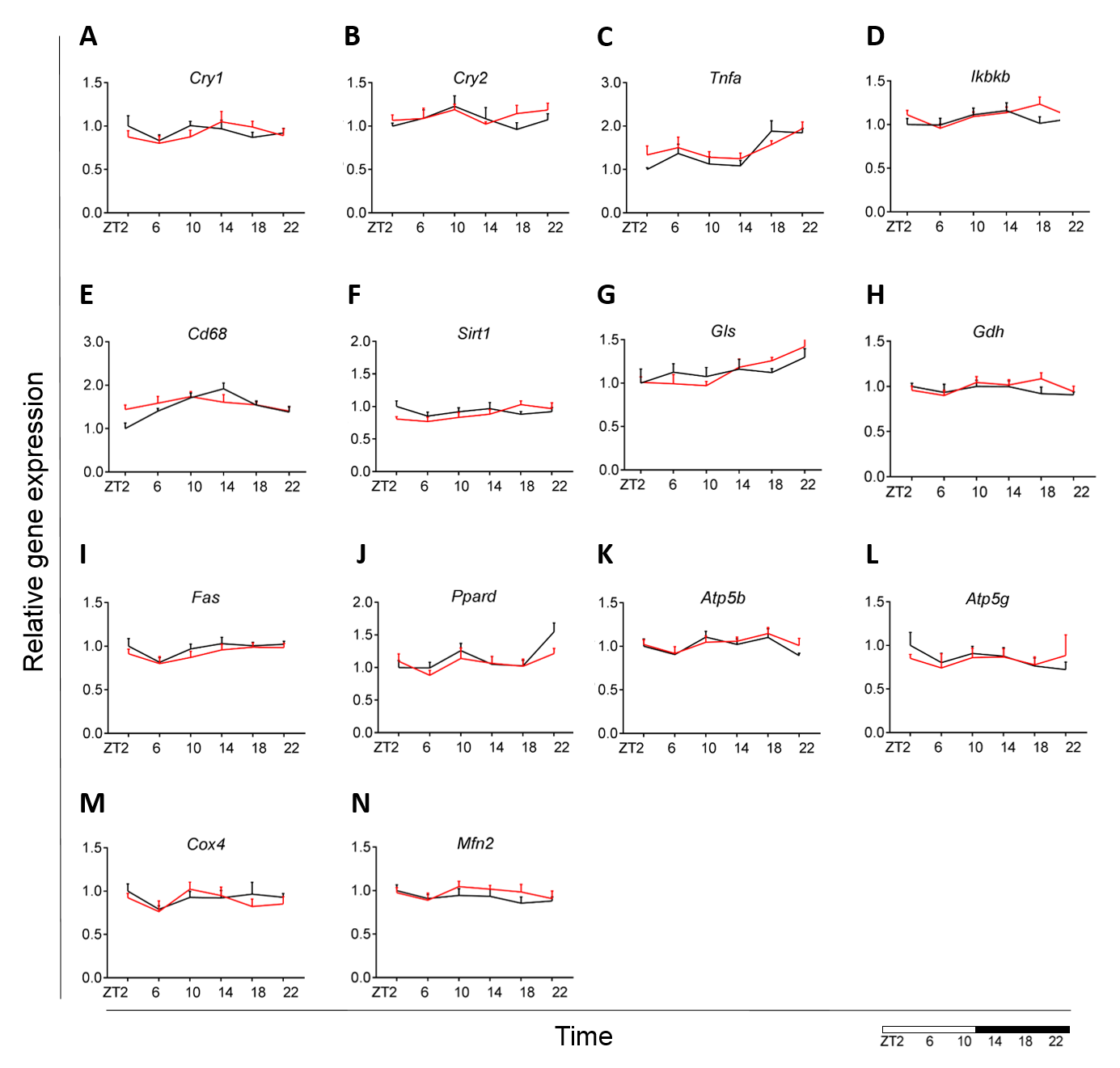
***

**Figure S1. Monocyte immunometabolism in obesity – additional data.** Relative gene expression of circadian genes *Cry1* (**A**) and *Cry2* (**B**); innate immunity genes *Tnfa* (**C**) and *Ikbkb* (**D**), phagocytic indicator gene *Cd68* (**E**), *Sirt1* gene (**F**); glutamate substrate utilization genes *Gls* (**G**) and *Gdh* (**H**); fatty acid synthesis gene *Fas* (**I**) and fatty acid sensing gene *Ppard* (**J**); mitochondria bioenergetics genes *Atp5b* (**K**), *Atp5g* (**L**) and *Cox4* (**M**) and mitochondria dynamics gene *Mfn2* (**N**) in HFD-fed rats (red) compared to Chow-fed controls (black) evaluated at six time points, starting at ZT2. Data are presented as means ± SEM. Statistical significance was determined using Two-way ANOVA effect for *Interaction*, *Diet* and *Time* (ZT); Student t-test is used for diet effect within a separate time point (p<0.05*). Scale **(bottom right)** represents light (ZT0-12) and dark (ZT12-24) phase.

***Figure S2. Average 24h food intake***


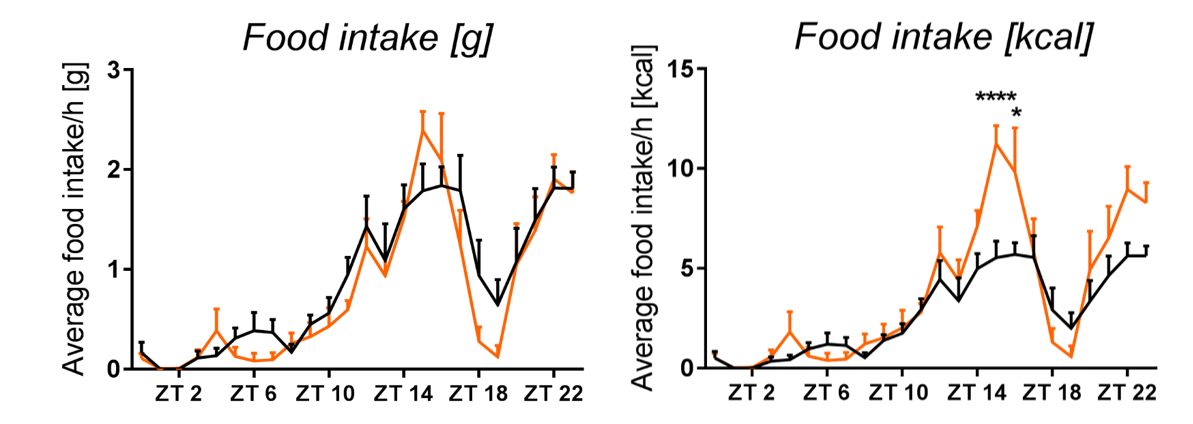


**Figure S2. Average time-of-day food intake per hour.** Graphs represented show a re-analysis of food intake data from metabolic cages for rats fed a HFD (4.7kcal/g) or control diet (3.1 kcal/g) (63). Data show average food intake per hour starting at ZT0. Data are presented as food intake in gram (p_int_ = 0.7440, p_time_ <0.0001, p_diet_ = 0.1001) **(left)** and kcal (p_int_ = 0.0033, p_time_ <0.0001, p_diet_ = 0.0003) **(right)**, as 24h mean of 48h measurement for control (black) and HFD (orange) groups (n=8). Data are presented as means ± SEM. Statistical significance was determined using Two-way ANOVA effect for *Interaction*, *Time* (ZT) and *Diet*; Sidak’s multiple comparison test was used to compare the effect of diet for each time point (p<0.05*; p<0.0001****).
